# Clinical Utility of LC3 and p62 Immunohistochemistry in Diagnosis of Drug-Induced Autophagic Vacuolar Myopathies: A Case-Control Study

**DOI:** 10.1371/journal.pone.0036221

**Published:** 2012-04-27

**Authors:** Han S. Lee, Brianne H. Daniels, Eduardo Salas, Andrew W. Bollen, Jayanta Debnath, Marta Margeta

**Affiliations:** 1 Department of Pathology, University of California San Francisco, San Francisco, California, United States of America; 2 College of Osteopathic Medicine, Touro University California, Vallejo, California, United States of America; University of Campinas, Brazil

## Abstract

**Background:**

Some patients treated with chloroquine, hydroxychloroquine, or colchicine develop autophagic vacuolar myopathy, the diagnosis of which currently requires electron microscopy. The goal of the current study was to develop an immunohistochemical diagnostic marker for this pathologic entity.

**Methodology:**

Microtubule-associated protein light chain 3 (LC3) has emerged as a robust marker of autophagosomes. LC3 binds p62/SQSTM1, an adapter protein that is selectively degraded via autophagy. In this study, we evaluated the utility of immunohistochemical stains for LC3 and p62 as diagnostic markers of drug-induced autophagic vacuolar myopathy. The staining was performed on archival muscle biopsy material, with subject assignment to normal control, drug-treated control, and autophagic myopathy groups based on history of drug use and morphologic criteria.

**Principal Findings:**

In all drug-treated subjects, but not in normal controls, LC3 and p62 showed punctate staining characteristic of autophagosome buildup. In the autophagic myopathy subjects, puncta were coarser and tended to coalesce into linear structures aligned with the longitudinal axis of the fiber, often in the vicinity of vacuoles. The percentage of LC3- and p62-positive fibers was significantly higher in the autophagic myopathy group compared to either the normal control (p<0.001) or the drug-treated control group (p<0.05). With the diagnostic threshold set between 8% and 15% positive fibers (depending on the desired level of sensitivity and specificity), immunohistochemical staining for either LC3 or p62 could be used to identify subjects with autophagic vacuolar myopathy within the drug-treated subject group (p≤0.001).

**Significance:**

Immunohistochemistry for LC3 and p62 can facilitate tissue-based diagnosis of drug-induced autophagic vacuolar myopathies. By limiting the need for electron microscopy (a time consuming and costly technique with high specificity, but low sensitivity), clinical use of these markers will improve the speed and accuracy of diagnosis, resulting in significantly improved clinical care.

## Introduction

Macroautophagy (hereafter called autophagy) is an evolutionarily conserved mechanism for degradation of cytoplasmic components, which at baseline contributes to cellular homeostasis by enabling routine protein and organelle turnover (reviewed in [1]–[3]). During periods of increased nutrient requirements, autophagy induction is critical for survival [4], [5]. Autophagy can also be regulated by non-metabolic factors such as oxidative stress, infection, and accumulation of aggregated proteins. Recently, it has been recognized that autophagy plays an important role in the pathogenesis of diverse diseases, including neurodegenerative, neoplastic, infectious, inflammatory, and neuromuscular conditions [1], [3].

Distinct from the ubiquitin-proteasome system, the process of autophagy occurs through a multi-step mechanism regulated by ATG (AuTophagy Gene) proteins [1]–[3]. Early steps include the formation of an isolation membrane (the phagophore), which engulfs proteins and organelles destined for degradation. Closure of the phagophore produces a membrane-bound vacuole (the autophagosome), which moves along microtubules and fuses with the lysosome to form the autolysosome (where the material is ultimately degraded). Microtubule-associated protein 1 light chain 3 (LC3), a mammalian orthologue of yeast ATG8, is commonly used as a marker of autophagosome formation [6], [7]. Normally cytosolic in its precursor form (LC3-I), LC3 undergoes proteolytic cleavage of the C-terminal end upon autophagy induction, resulting in exposure of a glycine residue and subsequent lipidation with a phosphatidylethanolamine group. This modified form, termed LC3-II, is associated with autophagic membranes and is preferentially detected by LC3 immunohistochemistry. LC3-II also binds p62/SQSTM1, an adapter protein that targets ubiquitinated protein aggregates for lysosomal degradation and is selectively degraded via autophagy [8]. Because LC3-II and p62 are both degraded in the autolysosome, the lysosomal-dependent turnover of these proteins has emerged as a measure of bona fide autophagic proteolysis, which is commonly termed autophagic flux. Specifically, the accumulation of LC3-II-labeled autophagosomes and/or p62 aggregates is a robust marker of autophagic flux inhibition at any point beyond autophagosome formation [7].

Autophagy inhibition plays a key role in the pathogenesis of several inherited myopathies including Danon disease, X-linked myopathy with excessive autophagy (XMEA), and infantile autophagic vacuolar myopathy [9], [10], all of which are characterized by accumulation of autophagic vacuoles. While pathogenesis of these disorders is still being elucidated, the overarching defect seems to involve lysosomal function. For example, Danon disease mutations involve the gene encoding lysosome-associated membrane protein 2 (LAMP2), a protein thought to play a role in autophagosome - lysosome fusion [11]. Similarly, XMEA is caused by mutations in VMA21 (a chaperone for lysosomal V-ATPase) that result in the impairment of lysosomal acidification (Berge Minassian, personal communication). While inherited autophagic vacuolar myopathies are quite rare, autophagic vacuolar myopathies caused by autophagy-inhibiting drugs (such as chloroquine, its analog hydroxychloroquine, and colchicine) are much more common [12]–[15]. Chloroquine and hydroxychloroquine accumulate within lysosomes and are thought to block autophagy through elevation of intralysosomal pH and inhibition of lysosomal enzymes [16], [17]. In addition, decreased autophagosome - lysosome fusion has been observed in cell culture models of chloroquine toxicity [18]. Colchicine, on the other hand, is a well-established microtubule-disrupting agent. Hence, colchicine likely blocks autophagy by disrupting the movement of autophagosomes and lysosomes along microtubules [14]. Consistent with this idea, reduced exocytosis of lysosomal contents has been observed following colchicine administration [19]. At the present, autophagy-inhibiting drugs are clinically used for treatment of malaria (chloroquine), rheumatologic disease (hydroxychloroquine), and gout (colchicine). In addition, multiple ongoing clinical trials are assessing the effectiveness of autophagy inhibitors as adjuvant cancer chemotherapy (reviewed in [20]–[23]). Thus, the incidence of drug-induced autophagic vacuolar myopathy can only be expected to rise in the future.

Currently, definitive diagnosis of autophagic vacuolar myopathies requires ultrastructural demonstration of autophagic vacuoles in a muscle biopsy, thus necessitating use of electron microscopy – a time-consuming and costly technique with a large possibility for sampling error. Development of an immunohistochemical method for detection of autophagosome accumulation thus has the potential to increase both the speed and accuracy of diagnosis, resulting in significant improvement in clinical care. Here, we show that immunohistochemistry for LC3 and p62 can be used to diagnose drug-induced autophagic vacuolar myopathies, obviating the need for electron microscopy in the great majority of clinically relevant scenarios.

## Methods

### Ethics Statement

Study design was reviewed and approved by the University of California San Francisco (UCSF) Committee on Human Research (CHR). Given the non-invasive nature of the study and a minimal potential for harm to study participants, the informed consent requirement was waived by the CHR. No individually identifiable patient data is presented in this report.

### Objectives

The objective of this study was to determine whether immunohistochemistry for LC3 and/or p62 can be used as a diagnostic tool for the diagnosis of drug-induced autophagic vacuolar myopathies.

### Participants

To identify cases of autophagic vacuolar myopathy related to colchicine, chloroquine or hydroxychloroquine use, we performed a computerized search of the UCSF neuropathology case database spanning the interval between 1990 and 2010; potential drug-treated control cases within the same time span were identified based on the history of the relevant drug use and the absence of autophagic vacuolar myopathy diagnosis (but were allowed to have other pathologic findings; see Table 1 for details). All subjects had a history of either colchicine or hydroxychloroquine treatment (no chloroquine-treated patients were identified in the database search). Normal controls were selected from a larger pool of muscle biopsies characterized by (1) lack of pathologic findings and (2) no history of autophagy inhibitor use. Availability of the archival formalin-fixed, paraffin-embedded (FFPE) and glutaraldehyde-fixed, Epon-embedded material were additional criteria for inclusion in the study. To ensure accurate classification of patients into “autophagic myopathy" and “drug-treated control" groups, blinded review of electron microscopy images was performed by two Board-certified neuropathologists (MM and AB) for all drug-treated cases; if ultrastructural analysis was not a part of the original diagnostic work-up, electron microscopy was performed on the original material as part of the study (see below). A minimum of 10 electron micrographs, taken by trained electron microscopy technicians, was reviewed for each case. Autophagic vacuolar myopathy was diagnosed based on ultrastructural identification of at least 15 definitive autophagic vacuoles in the image set (although many cases had more than 50); drug-treated control cases showed either none or very few autophagic vacuoles, with largest number (4) identified in specimen #12. Only the cases with consensus diagnosis were included in the study (1 case with borderline findings and 2 cases without consensus diagnosis were excluded). For the 7 drug-treated control subjects, pathologic diagnoses reported in Table 1 were made following the review of original light microscopy slides including the following: hematoxylin and eosin (H&E) stain of the FFPE material; H&E, modified trichrome, ATPase (pH 9.2), NADH reductase, and SDH stains of the frozen material; and Toluidine Blue stain of the glutaraldehyde-fixed material. Given that case and control group assignment was based solely on history of drug use and morphologic criteria, no attempt was made to match participants by age, sex, or other demographic variables.

**Table 1:** Study subject characteristics.

Subject ID	Age	Sex	Drug	Treatment Duration	Pathologic Diagnosis	LC3 (% positive fibers)	p62 (% positive fibers)
**Normal Control Group**							
1	52	F	None	NA	Normal	0.0	0.0
2	67	F	None	NA	Normal	0.0	0.0
3#	83	F	None	NA	Normal	0.0	0.0
4#	56	F	None	NA	Normal	0.0	0.0
5	53	M	None	NA	Normal	0.0	0.0
6	57	M	None	NA	Normal	0.0	0.0
7	60	M	None	NA	Normal	0.0	0.0
8#	64	F	None	NA	Normal	0.0	1.0
9	48	M	None	NA	Normal	0.0	0.0
10	32	M	None	NA	Normal	0.0	0.0
**Drug-Treated Control Group**							
11#	76	M	Colchicine	Unknown	Necrotizing myopathy	2.0	3.1
12	32	F	Hydroxychloroquine	3 years	Necrotizing myopathy*	13.0	10.5
13#	58	F	Hydroxychloroquine	≥20 days	Inflammatory myopathy	2.5	4.3
14#	33	M	Hydroxychloroquine	1.5 years; stopped; then 1 month	Normal	0.3	0.2
15	65	F	Hydroxychloroquine	≥2 months	Neurogenic changes	4.0	2.0
16#	44	F	Colchicine	Unknown	Neurogenic changes**	1.8	1.5
17#	47	F	Hydroxychloroquine	≤1 year	Normal	0.8	0.3
**Autophagic Myopathy Group**							
18	73	M	Colchicine	≤2 years	Autophagic myopathy	64.0	86.0
19§	58	M	Colchicine	Unknown	Autophagic myopathy	22.5	14.8
20	81	F	Colchicine	Unknown	Autophagic myopathy	78.0	83.5
21	84	M	Colchicine	10 days	Autophagic myopathy	58.5	65.0
22	80	M	Colchicine	“Chronic"	Autophagic myopathy	13.0	21.0
23	72	F	Hydroxychloroquine	Unknown	Autophagic myopathy	56.5	53.0
24	33	F	Hydroxychloroquine	1 month	Autophagic myopathy	18.0	12.5
25	79	M	Colchicine	“Chronic"	Autophagic myopathy	79.0	79.5
26	79	M	Colchicine	Unknown	Autophagic myopathy	95.0	93.0
27	29	M	Hydroxychloroquine	>1 year	Autophagic myopathy	12.5	25.5

# § To minimize sampling error due to scant (^#^) or patchy (^§^) positivity, a total of 600 (rather than 200) fibers was counted.

* Scattered basophilic cores were present.

** Multiple rimmed vacuoles were present.

NA Not applicable.

### Procedures

#### Electron microscopy

Ultrathin (80 nm) sections of the original glutaraldehyde-fixed, Epon-embedded tissue were stained with 2% uranyl acetate at the UCSF Electron Microscopy Core Lab. Sections were subsequently examined in a JEOL 1400 transmission electron microscope at 120 kV. Images were recorded with a Gatan SC1000 CCDE camera.

#### Immunohistochemistry

Immunoperoxidase staining for LC3 (mouse monoclonal antibody, clone 5F10, Nanotools) and p62/SQSTM1 (guinea pig polyclonal antibody, Progen Biotechnik) was performed using Ventana Benchmark XT automated slide preparation system at the UCSF Brain Tumor Research Center Tissue Core. Immunoperoxidase staining was performed on the FFPE tissues of all subjects and on frozen tissue from 11 representative subjects (2 normal controls, 3 drug-treated controls, and 6 autophagic myopathy cases). Tissue sections (4–5 µm thickness) were deparaffinized (EZ-Prep, Ventana Medical Systems, at 75°C) followed by antigen-retrieval (Cell Conditioning 1, Ventana Medical Systems, at 95–100°C). Antibodies were incubated at room temperature for 2 h, at 1∶100 dilutions. Antibody staining was developed using the UltraView Universal DAB detection system (Ventana Medical Systems), and accompanied by hematoxylin counterstain. Frozen sections were fixed in ice cold 100% acetone at −20°C for 5 min prior to immunostaining as described above; no antigen retrieval was required for frozen sections.

#### Quantification

Quantification was performed on immunostained sections of FFPE material using a bright-field light microscope, with the investigator blinded to group assignment of each subject. Prior to counting, each slide was viewed at low (2×–20×) and high power (40×) to determine whether positive fibers were present scarcely or in abundance. Muscle fibers containing the characteristic central inclusion, rimmed vacuole, or punctate staining pattern were counted as positive, while fibers lacking such staining were counted as negative. A total of 200 fibers/slide was counted in specimens with abundant positivity, while a total of 600 fibers/slide was counted in specimens with scarce or patchy positivity (to reduce the sampling error). Tissue on the slide was divided into quadrants and randomly selected non-overlapping fields were counted at high power in each quadrant until the total count was reached. The results were recorded as a percentage (the number of positive fibers divided by the total number of fibers counted).

#### Imaging

Images were taken with a DP72 digital camera on a BX41 bright-field light microscope using cellSens Entry 1.4 software (all by Olympus Corp) and were edited with Adobe Photoshop CS5 Version 12.0.3.

#### Immunoblot analysis

Immortalized ATG7+/+ and ATG7−/− mouse embryonic fibroblasts [4], a gift from Dr. Masaaki Komatsu (Tokyo Metropolitan Institute), were cultured in DMEM containing 25 mM glucose (Invitrogen) supplemented with 10% FBS, penicillin, and streptomycin. Following 8 h treatment with 30 µM chloroquine (dissolved directly in growth medium), fibroblasts were lysed in RIPA lysis buffer (Sigma-Aldrich; catalog number R0278) plus 10 mM NaF, 10 mM β-glycerophosphate, 1 mM Na_3_VO_3_, 10 nM calyculin A, and protease inhibitors. Snap-frozen human muscle biopsy tissue was thawed on ice in RIPA lysis buffer with aforementioned supplements and lysosomal inhibitors E-64d and pepstatin A (10 µg/ml each); subsequently, samples were sonicated at 2 W on ice using a Fisher Scientific 60 Sonic Dismembrator until homogenized. Crude homogenates were cleared by centrifugation at 14,000 rpm for 15 min at 4°C. Following measurement of protein concentration with a BCA protein assay (Thermo), cleared homogenates were boiled in SDS sample buffer, resolved using SDS- PAGE (15–50 µg of total protein per lane), and transferred to polyvinylidene difluoride membranes. Membranes were blocked in PBS +0.1% Tween 20 with 5% nonfat dry milk, incubated with the primary antibodies overnight at 4°C, washed, incubated with horseradish peroxidase-conjugated secondary antibodies, and analyzed by enhanced chemiluminescence. Primary antibodies included anti-LC3 rabbit polyclonal antibody generated against a peptide corresponding to the N-terminus common to human, mouse, and rat LC3 (Fung et al., 2008), guinea pig polyclonal anti-p62/SQSTM1 antibody (Progen Biotechnik), and mouse monoclonal anti-GAPDH antibody (Chemicon).

### Statistical methods

Data were analyzed with GraphPad Prism 5 statistical software. For between-group comparison of the demographic and treatment variables, we used one-way ANOVA with post-hoc Bonferroni test (age) or chi-square test (sex and drug treatment). LC3 and p62 positivity data were not normally distributed; thus, between-group comparison for all three study groups was performed by Kruskal-Wallis one-way ANOVA on ranks. To calculate diagnostic threshold values with optimal sensitivity and specificity, receiver operating characteristic (ROC) analysis was performed on the two drug-treated groups (autophagic myopathy vs. drug-treated control group). All tests were 2-tailed with α = 0.05.

## Results

### Histologic and ultrastructural findings

**Figure 1:**
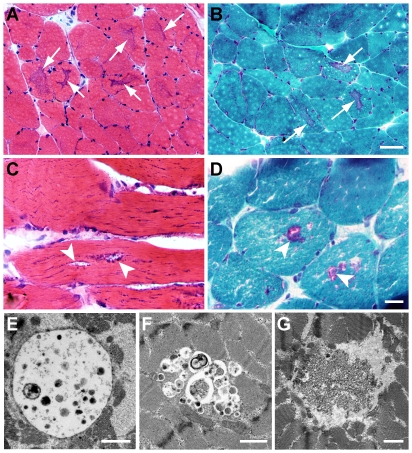
Histologic and ultrastructural patterns in autophagic vacuolar myopathy. **A and B.** Sharply defined, central areas of dark staining (“basophilic cores"; arrows) seen on H&E- (A) and trichrome- (B) stained sections of frozen material (subject #22). **C and D.** Rimmed vacuoles (arrowheads) seen on H&E- (C) and trichrome- (D) stained sections of frozen material (subject #23) **E–G.** Autophagic vacuoles with membrane-bound (E; subject #22) or “free-floating" (F; subject #27) cellular debris on electron microscopy. Curvilinear bodies in a hydroxychloroquine-treated subject (G; subject #23). Scale bars: A and B, 50 µM; C and D, 20 µM; E–G, 1 µm.

On light microscopy, we identified several histologic patterns suggestive (although not diagnostic) of autophagic vacuolar myopathy. The most common finding was the presence of sharply delineated areas of central dark staining, which we termed “basophilic cores". Basophilic cores were best visualized on H&E (Fig. 1A), trichrome (Fig. 1B), and NADH reductase stains (supplemental Fig. S1-A) of frozen material; their vacuolated nature was highlighted by Toluidine Blue stain of Epon-embedded material (supplemental Fig. S1-B). Basophilic core-like structures have been observed in both human and animal muscle following colchicine or chloroquine treatment and have been described as areas of myofibrillar disarray [24], [25], NADH-positive alterations in intermyofibrillar network [26], and central basophilia [19]. Interestingly, similar “hematoxylin-positive structures" were seen in autophagy deficient (ATG7−/−) murine muscle, but only following denervation [27]. In the current study, basophilic cores were seen in 5 of 10 autophagic myopathy cases and in 1 of 7 drug-treated controls (subject #12). Another relatively common finding was the presence of classic rimmed vacuoles (arrowheads, Figs. 1C and 1D), which were indistinguishable from rimmed vacuoles seen in inclusion body myositis or inherited inclusion body myopathy. In the current study, this pattern was seen in 3 of 10 autophagic myopathy cases and in 1 of 7 drug-treated controls. Finally, some autophagic myopathy cases showed entirely non-specific myopathic features, with vague vacuolization difficult to distinguish from processing and/or preservation artifacts (not shown). There was no correlation between the type of drug treatment and the histologic pattern seen. On electron microscopy, we identified autophagic vacuoles containing either membrane-bound (Fig. 1E) or “free-floating" (Fig. 1F) cellular debris; there was no correlation between the presence of either of these ultrastructural patterns and histologic findings described above or the type of drug treatment the subject received. Curvilinear bodies (Fig. 1G) have been documented in many cases of hydroxychloroquine myopathy [28], [29]; in our autophagic myopathy group, they were present in 2 of 3 subjects treated with hydroxychloroquine and in 0 of 7 subjects treated with colchicine.

### LC3 and p62 immunohistochemistry

**Figure 2:**
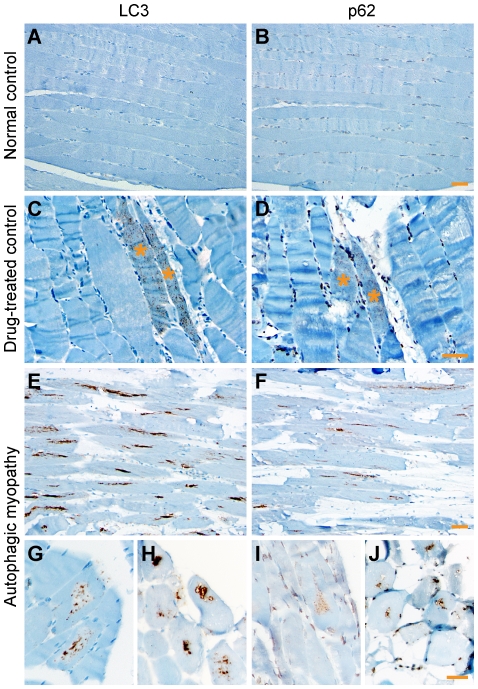
LC3 and p62 immunohistochemistry on FFPE material. **A and B.** Lack of sarcoplasmic staining in a normal control subject (#8). With p62, there was background nuclear positivity. **C and D.** Rare fibers (asterisks) with finely punctate staining in a drug-treated control subject (#11). **E–J.** Moderate to frequent LC3- and p62-positive fibers in autophagic myopathy subjects. On longitudinal sections (E and F; subject #23), the staining was linear, aligned with the longitudinal axis of the fiber, and often centrally located. On cross sections, the staining was coarsely punctate (G and I; subject #22) or confluent, often lining vacuole rims (H and J; subject #23). Scale bars, 50 µM.

Immunohistochemistry for LC3 and p62 was performed on FFPE tissue. In normal control samples, there was no sarcoplasmic staining (Figs. 2A and 2B); nuclear positivity, seen in p62-stained sections, is typical for the specific anti-p62 antibody used and was previously observed in human brain sections [30]. In samples from the drug-treated control group, LC3 and p62 positivity was seen in rare fibers that typically showed a finely punctate staining pattern (Figs. 2C and 2D). Occasionally, we observed coarser staining coalescing around a proto-vacuole (supplemental Fig. S2). In contrast, samples from the autophagic myopathy group showed moderate to frequent LC3- and p62-positive fibers characterized by coarsely punctate staining pattern (Figs. 2G and 2I) that tended to coalesce, resulting in zones of increased staining running linearly along the longitudinal axis of the fiber and most commonly located in the fiber center (Figs. 2E and 2F). LC3 and p62 staining was also seen lining the rimmed vacuoles (Figs. 2H and 2J) and, less frequently, at the periphery of fibers or under the sarcolemma (not shown). When LC3 and p62 immunohistochemistry was performed on the frozen tissue, similar staining patterns were observed (supplemental Fig. S3).

**Figure 3:**
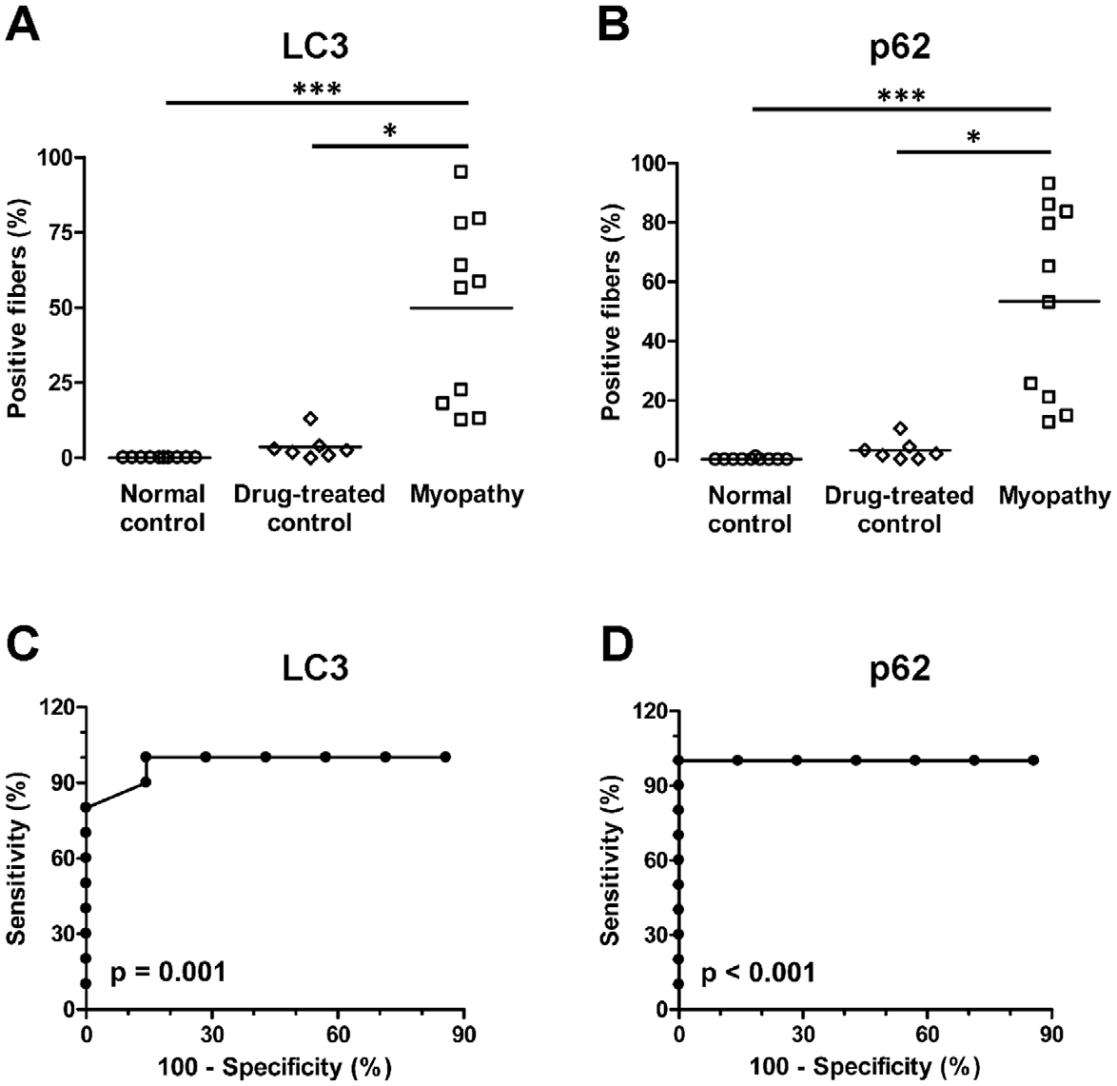
Quantification of LC3- and p62-positive fibers in FFPE material. **A and B.** The percentage of LC3- (**A**) and p62-positive (**B**) fibers was significantly higher in autophagic myopathy group (squares) than in either normal control (circles) or drug-treated control group (diamonds). Each study subject is represented with a symbol; the lines indicate group means. ***, p<0.001; *, p<0.05. **C and D.** ROC analysis indicates that quantitative immunohistochemistry for either LC3 (C) or p62 (D) can successfully differentiate autophagic myopathy from control cases among drug-treated subjects.

To statistically compare the degree of LC3 and p62 positivity between the three experimental groups, we quantified the percentage of LC3- and p62-positive muscle fibers in FFPE sections; data for individual subjects are shown in Table 1. The percentage of LC3-positive fibers was significantly higher in the autophagic myopathy group (median 57.5%, SD 30.8%) compared to either the normal control group (median 0.0%, SD 0.0%; p<0.001) or the drug-treated control group (median 1.8%, SD 4.5%; p<0.05) (Fig. 3A; Kruskal-Wallis one-way ANOVA on ranks). Similar results were seen with p62 staining: the percentage of p62-positive fibers was significantly higher in the autophagic myopathy group (median 59.0%, SD 32.2%) compared to either the normal control group (median 0.0%, SD 0.3%; p<0.001) or the drug-treated control group (median 2.0%, SD 3.6%; p<0.05) (Fig. 3B; Kruskal-Wallis one-way ANOVA on ranks). To assess the diagnostic value of LC3 and p62 immunohistochemistry, we performed ROC (receiver-operator characteristic) curve analysis using the data from the two drug-treated groups (Figs. 3C and 3D). ROC analysis showed that either test can effectively distinguish autophagic myopathy from drug-treated control specimens (p-value for area under ROC curve: LC3, p = 0.001; p62, p<0.001). For LC3, there was a small trade-off between sensitivity and specificity, with a threshold value of 8.3% resulting in 100% sensitivity and 85.7% specificity, and a threshold value of 15.5% resulting in 80% sensitivity and 100% specificity. For p62, the optimal threshold value was 11.5% (100% sensitivity and 100% specificity).

### LC3 and p62 immunoblotting

**Figure 4:**
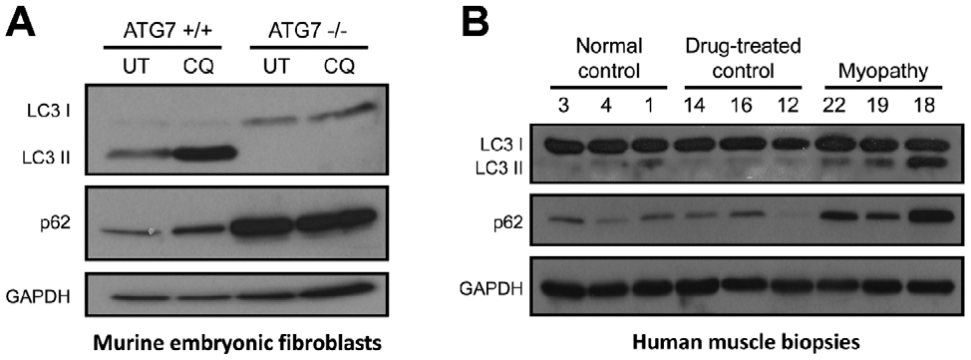
Immunoblotting confirms the specificity of LC3 and p62 immunohistochemistry. **A.** Wt (ATG7+/+) and autophagy-deficient (ATG7−/−) mouse embryonic fibroblasts (MEFs) were used as positive control. In wt MEFs, 8 h treatment with 30 µM chloroquine (CQ) increased the level of LC3-II and p62 proteins compared to the untreated control (UT); LC3-I was barely detectable in either sample. In autophagy-deficient MEFs, the level of LC3-I and p62 was high at baseline and did not change following CQ treatment; LC3-II was undetectable in both samples. GAPDH was used as a loading control. **B.** In subjects from the autophagic myopathy group, LC3-II and p62 protein level was increased relative to subjects from either normal or drug-treated control groups. LC3-I protein level was equally high in all samples, suggesting that this isoform is not detected by LC3 immunohistochemistry. Each lane contains sample from a different study subject, with subject ID numbers indicated on top. GAPDH was used as a loading control.

To confirm that LC3 staining in autophagic myopathy samples reflects an increase in the autophagosome-bound LC3-II rather than the cytoplasmic LC3-I, we performed immunoblotting for LC3 on a representative subset of muscle biopsies; ATG7 +/+ and −/− mouse embryonic fibroblasts (MEFs) were used as a positive control. Immunoblotting for p62 was performed in parallel on both sets of samples (Fig. 4). As expected, chloroquine treatment of ATG7+/+ MEFs resulted in an increase in LC3-II and p62 protein level. In contrast, autophagy-deficient ATG7−/− MEFs showed no LC3-II formation and a large increase in the level of p62 protein, both of which were independent of chloroquine treatment (Fig. 4A). Immunoblotting of human muscle samples showed results similar to wt (ATG7+/+) MEFs: protein level of LC3-II and p62 was higher in subjects from the autophagic myopathy group than in subjects from either the normal control or drug-treated control groups (Fig. 4B). Interestingly, LC3-II and p62 protein level was lower in samples #19 and #22 (which showed LC3 and p62-positive fibers in 13.0–22.5% range) than in sample #18 (which had 64.0% LC3-positive and 86.0% p62-positive fibers). Thus, there is a good correspondence between LC3-II and p62 protein level on immunoblotting and the percentage of positive fibers on immunostaining, confirming both the specificity and diagnostic utility of LC3 and p62 immunohistochemistry in this clinical context.

### Demographics

Participant assignment to normal control, drug-treated control, and autophagic myopathy study groups was based solely on morphologic criteria and history of autophagy inhibitor use (see *Methods* for details). While it is never possible to definitively prove the drug etiology of autophagic vacuolar myopathy, drug toxicity is the most likely explanation for changes seen in this group of subjects given the (1) positive history of relevant drug use, (2) negative family history, (3) very low prevalence of inherited autophagic vacuolar myopathies in the general population, (4) subject age and sex, and (5) the lack of histologic features (such as glycogen accumulation) that would suggest a different etiology.

There was no statistically significant difference in the mean age (57.2±13.3 [SD] vs. 50.7±16.4 vs. 66.8±20.2 years, respectively; p = 0.16) or sex distribution (50% vs. 71% vs. 30% female, respectively; p = 0.24) between the three study groups. Similarly, the type of drug treatment was not significantly different between the two drug-treated groups (29% colchicine for drug-treated control group vs. 70% colchicine for autophagic myopathy group; p = 0.09). However, this analysis is limited by the relatively small sample size; a larger study will be required to establish whether a trend for preponderance of male, colchicine-treated subjects in the autophagic myopathy relative to the drug-treated control group is biologically meaningful and whether sex and drug treatment are independent or dependent variables.

The length of drug treatment duration prior to biopsy was available in the clinical record of 5 of 7 drug-treated control subjects and 6 of 10 autophagic myopathy subjects (Table 1). Given the incompleteness of the dataset and variations in the reporting precision, statistical analysis of this parameter was not possible; however, both acutely- (3 months or less) and chronically- (1 year or more) treated subjects were present in each group.

No pathologic features were present in muscle biopsies from subjects included in the normal control group. In contrast, several different pathologies were present in the drug-treated control group (no pathologic findings [2 subjects]; necrotizing myopathy, not otherwise specified [2 subjects]; neurogenic change [2 subjects]; and inflammatory myopathy [1 subject]). Demographic, diagnostic, and drug treatment information for all study subjects is provided in Table 1.

## Discussion

Currently, definitive pathologic diagnosis of autophagic vacuolar myopathy requires electron microscopic identification of moderate to frequent well-developed autophagic vacuoles. In this study, we demonstrate that immunohistochemistry for LC3 and/or p62 can be used to detect autophagosome accumulation by light microscopy, thus providing a valuable diagnostic tool for this group of disorders.

In both autophagic myopathy and drug-treated control muscle, LC3 and p62 staining was largely punctate in nature. The punctate pattern of LC3 staining reflects the association of LC3-II with the membranes of early autophagosomes, whereas p62 puncta correspond to the accumulation of protein aggregates within early autophagic (LC3-positive) vesicles; hence, the increased punctate staining seen with these markers corresponds to autophagosome buildup [7], [31]. In drug-treated control specimens, the puncta were largely small and distributed evenly throughout the sarcoplasm (Figs. 2C and 2D). In autophagic myopathy specimens, on the other hand, the puncta were larger and primarily (although not exclusively) located in the center of the fiber, creating a linear structure aligned with the fiber's long axis. This linear pattern of staining may be related to the linear intermyofibrillar organization of microtubules (along which autophagosomes propagate) [19], and likely corresponds to the centrally-located zone of autophagic buildup that was detected in autophagy-deficient (ATG5−/−) murine skeletal muscle [32]. In addition to these qualitative differences in the pattern of staining between autophagic myopathy and drug-treated control samples, there was a significant difference in the percentage of LC3 and p62-positive fibers between the two drug-treated groups (no staining was observed in the normal control group). In autophagic myopathy subjects, staining was present in a large fraction of muscle fibers (12.5% to 95% for LC3; 12.5% to 93% for p62). In contrast, the majority of drug-treated control samples showed staining in less than 5% of muscle fibers, with only 1 of 7 specimens showing staining in up to 13% of fibers.

Based on the ROC analysis of our data, 100% specificity can be achieved by setting a diagnostic threshold at 15% LC3-positive muscle fibers. With this threshold, sensitivity is 80% (meaning that 20% of autophagic myopathies would be missed). In contrast, threshold of 8% LC3-positive fibers would achieve 100% sensitivity and 86% specificity (i.e., 14% of non-specific cases would be falsely diagnosed as autophagic vacuolar myopathy). Thus, using LC3 immunohistochemistry alone, a subset of cases with 8% to 15% of LC3-positive fibers would fall in the diagnostic “gray zone". For cases in this range, diagnostic accuracy can be improved by using additional diagnostic modalities, such as p62 immunohistochemistry and electron microscopy, and integrating the information gleaned from the standard histology and histochemistry stains (for example, the presence or absence of basophilic cores). In this context, it is worth noting that a single specimen from the drug-treated control group (#12, Table 1) showed a degree of staining (13% for LC3, 10.5% for p62) that overlapped with the range generally seen in the autophagic myopathy group. This specimen was designated a drug-treated control because only rare well-developed autophagic vacuoles were identified on ultrastructural examination. However, light microscopy showed scattered basophilic cores in the context of a mild necrotizing myopathy. Thus, it is possible that this case represents an instance of early and/or mild autophagic vacuolar myopathy that was missed on electron microscopy. (Ultrastructural examination has high specificity but low sensitivity, resulting in sampling bias and significant possibility of false negative results.) When this case is excluded from the ROC analysis, the optimal (100% sensitivity and 100% specificity) threshold value for diagnosis of autophagic vacuolar myopathy is 8% positive fibers on either LC3 or p62 immunohistochemistry.

In rodent models of chloroquine toxicity, concurrent denervation greatly contributes to the development of vacuolar pathology [33], [34]. Similarly, basophilic core-like structures were not present in the autophagy deficient (ATG7−/−) murine muscle at baseline, developing only after muscle denervation [27]. These findings raise the possibility that a concurrent neurogenic process contributes to the development of autophagic vacuolar myopathy in patients treated with chloroquine, hydroxychloroquine, or colchicine. Our data do not support this possibility: the drug-treated control group included two specimens (#15 and #16) with well-developed neurogenic changes, both of which showed a very low degree of LC3 and p62 labeling. However, a separate study will be required to definitively answer this question.

Rimmed vacuoles were detected in a subset of autophagic myopathy specimens both by standard histology (Figs. 1C and 1D) and on LC3 or p62 immunohistochemistry (Figs. 2H and 2J). However, rimmed vacuoles are not specific for autophagic vacuolar myopathies and can be seen in other disorders of skeletal muscle such as inclusion body myositis and several subtypes of muscular dystrophy. Indeed, positivity for either LC3 or p62 has already been noted in inclusion body myositis [35]–[37], although careful quantification and determination of proper diagnostic thresholds still needs to be done. Similarly, inherited autophagic vacuolar myopathies (such as Danon disease or XMEA) would be expected to be highly LC3 and p62 positive, but were not included in the current study because our archive does not include a sufficient number of well documented cases. While diagnostically helpful, neither LC3 nor p62 positivity is therefore pathognomonic for drug-induced autophagic vacuolar myopathies. To establish the correct diagnosis, positive LC3 and/or p62 staining needs to be correlated with the remainder of histologic findings and with full clinical history (including age at presentation, family history, and medication history).

The current study was not designed to detect differences in age, sex, or drug treatment distribution between the two drug-treated study groups. Nonetheless, we found that male, colchicine-treated subjects were more common in the autophagic myopathy group, while female, hydroxychloroquine-treated subjects were more common in the drug-treated control group. The differences we observed were not statistically significant; however, a study with 95% power to detect the effects of magnitude we observed (with a significance level of 0.05) would have needed to have 40 subjects in each group. Therefore, a larger study – ideally with a prospective design – will need to be performed in the future to establish whether certain types of patients are more vulnerable to development of drug-induced autophagic vacuolar myopathy.

In summary, we used specimens from human subjects treated with either hydroxychloroquine or colchicine to establish that immunohistochemical stains for LC3 and/or p62 are useful ancillary tools in pathologic diagnosis of drug-induced autophagic vacuolar myopathies. By limiting the need for electron microscopy, use of LC3 and/or p62 immunohistochemistry will decrease both the time required to establish the diagnosis and the false negative rate resulting from sampling bias, thus resulting in significantly improved clinical care.

### Limitations

The major strengths of the current study are (1) the use of pathologically well characterized subjects, (2) the inclusion of two control groups (normal controls and drug-treated controls), (3) the inclusion of drug-treated control subjects with abnormal muscle, mimicking clinically relevant scenarios for diagnostic test use, (4) the concordance of results across several experimental modalities (immunostaining of FFPE tissue vs. immunostaining of frozen tissue vs. immunoblotting of frozen tissue), and (5) the quantitative study design, which enabled calculation of sensitivity and specificity values for different diagnostic thresholds. The major limitations are (1) the under-sampling of the drug-treated control group, as patients with no symptoms are unlikely to undergo a muscle biopsy, and (2) a non-negligible probability that one (or more) drug-treated subjects were miss-assigned to the control group given the significant false negative rate of electron microscopy (the current “gold standard" method for diagnosis of autophagic vacuolar myopathies). As discussed above, these limitations would be expected to result in an elevated diagnostic threshold and artificial widening of the diagnostic “gray zone". Given that alternative treatments exist for both rheumatologic diseases and gout, it would thus be reasonable to use a fairly low diagnostic threshold (5% of LC3- or p62-positive fibers) for recommendation to discontinue hydroxychloroquine or colchicine therapy in a symptomatic patient with otherwise equivocal pathologic findings.

## Supporting Information

Figure S1 **Basophilic cores in an autophagic myopathy subject (#22).** **A.** On NADH histochemistry, basophilic cores (arrows) are centrally located, sharply demarcated, and darker than the rest of the fiber (in contrast to the central cores and mini-cores, which are lighter than the surrounding sarcoplasm). **B.** Toluidine Blue stain of the glutaraldehyde-fixed, Epon-embedded tissue shows that basophilic cores (arrows) contain numerous vacuoles. Scale bar, 50 µM.
(TIF)

Figure S2 **LC3 and p62 immunohistochemistry on FFPE sections from a drug-treated control subject (#16).** Coarsely punctate staining rims a proto-vacuole; this staining pattern was rare in drug-treated control subjects. Scale bar, 20 µM.
(TIF)

Figure S3 **LC3 and p62 immunohistochemistry on the frozen material.** **A and B.** No sarcoplasmic staining is seen in a drug-treated control subject (#14). **C and D.** Coarsely punctate sarcoplasmic staining is seen in an autophagic myopathy subject (#21). Focal sarcolemmal p62 positivity is seen in both control (B) and myopathy (D) subjects; given that no corresponding staining is present in LC3-stained sections (A and C), this likely represents a cryosection-specific staining artifact of the p62 antibody. Scale bar, 50 µM.
(TIF)
