# Supplementary figures and images for: Clinical Utility of LC3 and p62 Immunohistochemistry in Diagnosis of Drug-Induced Autophagic Vacuolar Myopathies: A Case-Control Study
Source: PLoS One. 2012 Apr 27;7(4):e36221. doi: 10.1371/journal.pone.0036221 (PMC3338695; doi:10.1371/journal.pone.0036221)

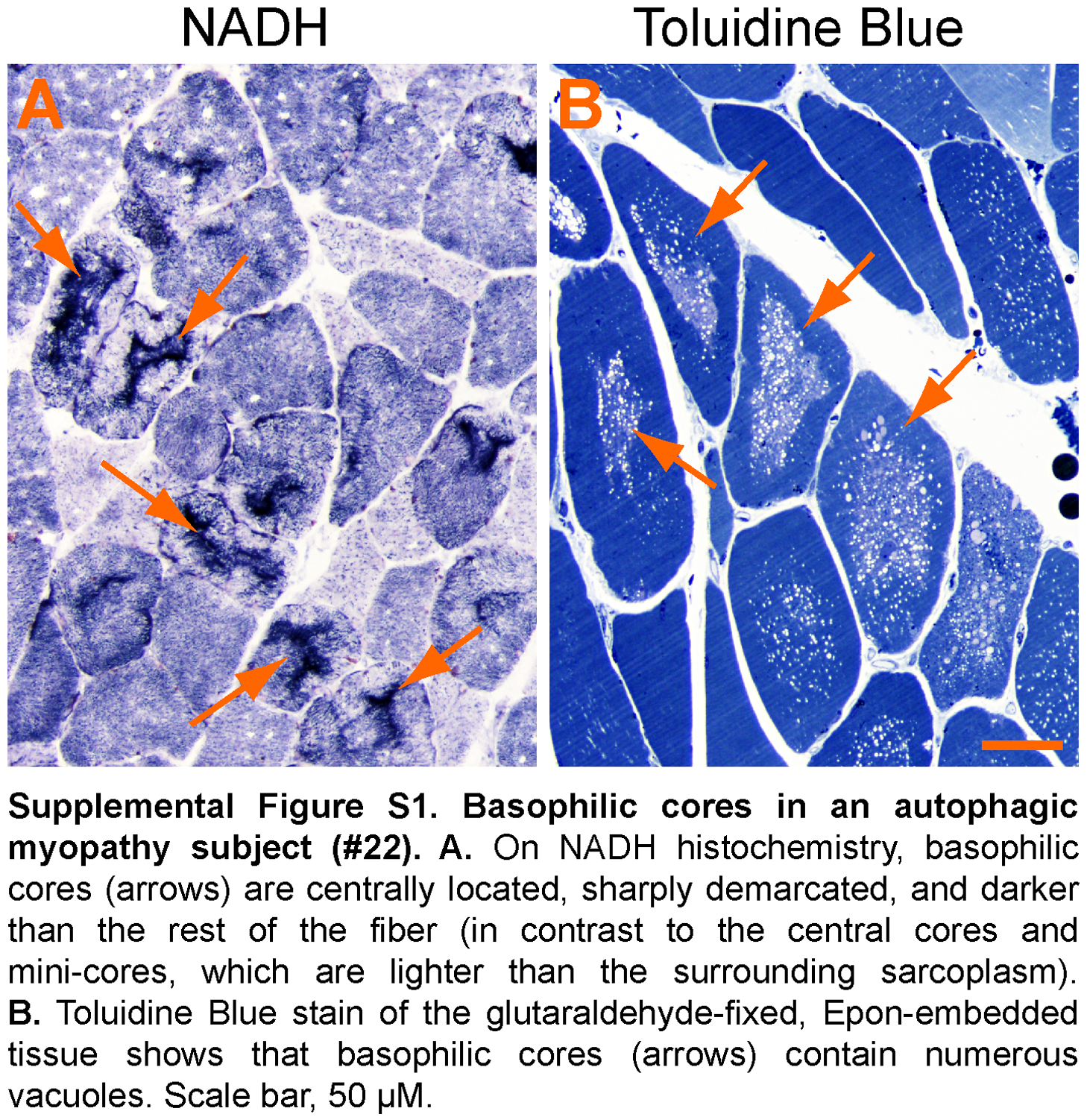

Supplement: Figure S1 — Basophilic cores in an autophagic myopathy subject (#22). A. On NADH histochemistry, basophilic cores (arrows) are centrally located, sharply demarcated, and darker than the rest of the fiber (in contrast to the central cores and mini-cores, which are lighter than the surrounding sarcoplasm). B. Toluidine Blue stain of the glutaraldehyde-fixed, Epon-embedded tissue shows that basophilic cores (arrows) contain numerous vacuoles. Scale bar, 50 µM. (TIF) [file pone.0036221.s001.tif]

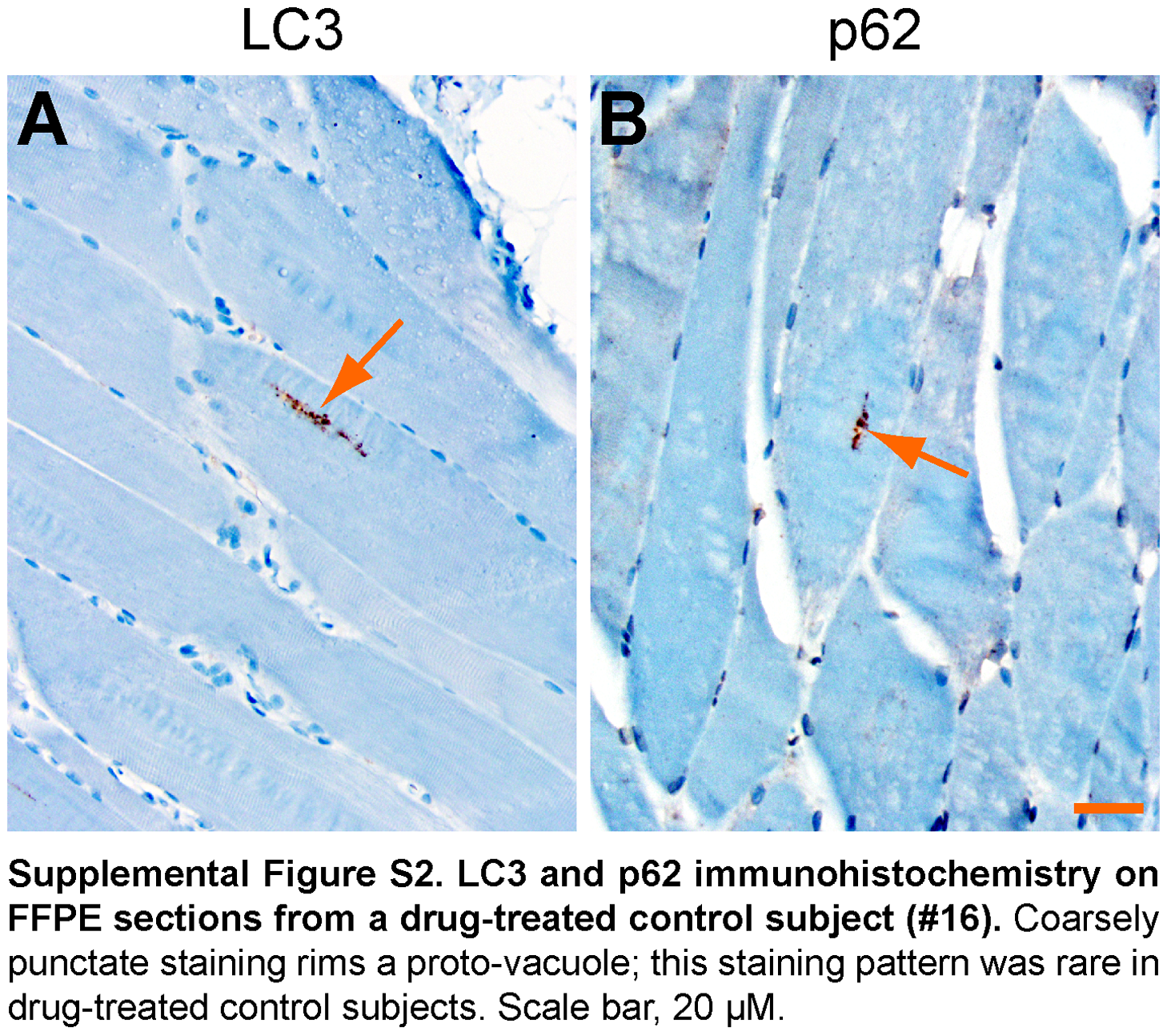

Supplement: Figure S2 — LC3 and p62 immunohistochemistry on FFPE sections from a drug-treated control subject (#16). Coarsely punctate staining rims a proto-vacuole; this staining pattern was rare in drug-treated control subjects. Scale bar, 20 µM. (TIF) [file pone.0036221.s002.tif]

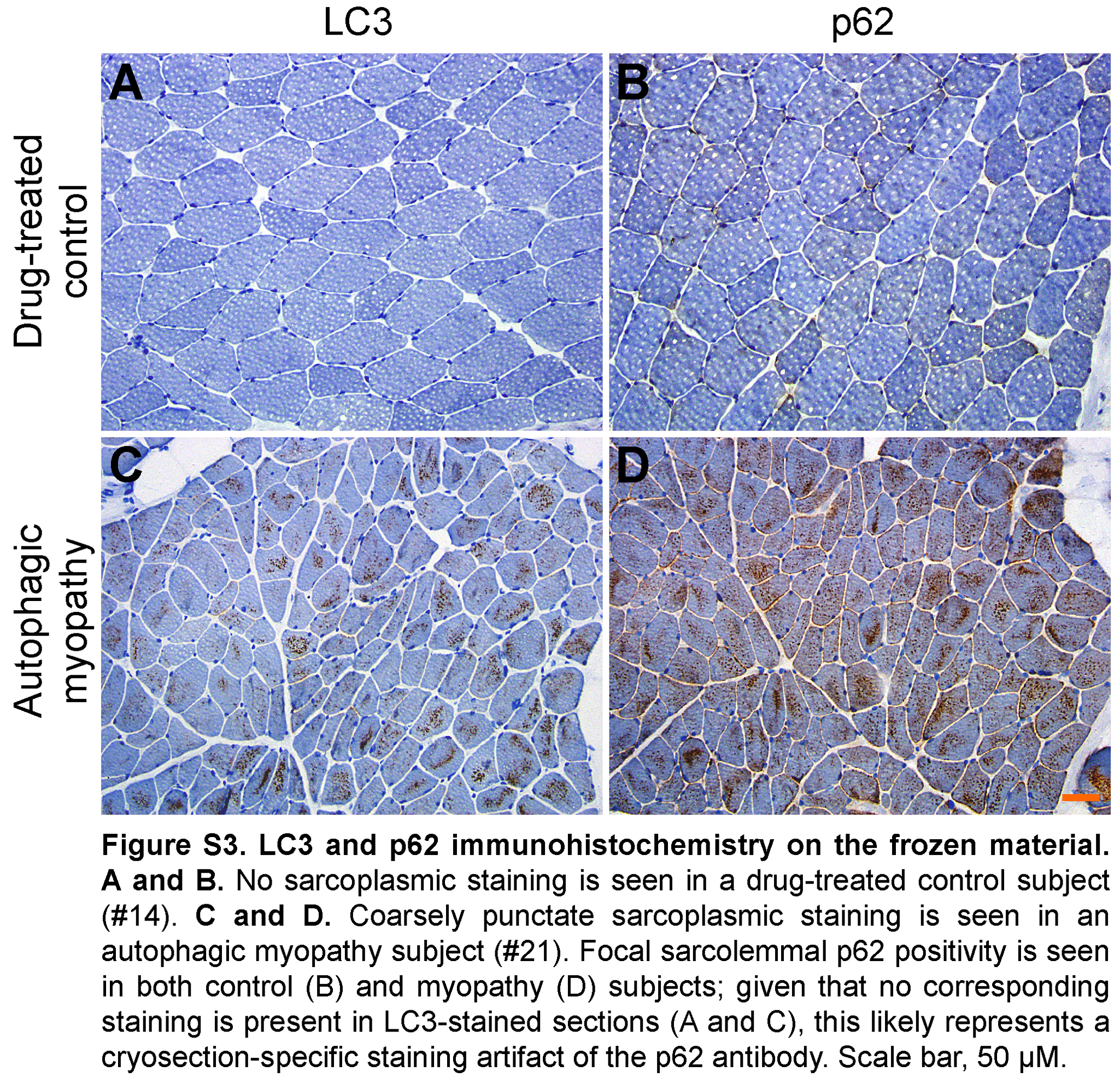

Supplement: Figure S3 — LC3 and p62 immunohistochemistry on the frozen material. A and B. No sarcoplasmic staining is seen in a drug-treated control subject (#14). C and D. Coarsely punctate sarcoplasmic staining is seen in an autophagic myopathy subject (#21). Focal sarcolemmal p62 positivity is seen in both control (B) and myopathy (D) subjects; given that no corresponding staining is present in LC3-stained sections (A and C), this likely represents a cryosection-specific staining artifact of the p62 antibody. Scale bar, 50 µM. (TIF) [file pone.0036221.s003.tif]
